# A viscoelastic–plastic deformation model of hemisphere-like tip growth in Arabidopsis zygotes

**DOI:** 10.1017/qpb.2024.13

**Published:** 2024-12-12

**Authors:** Zichen Kang, Tomonobu Nonoyama, Yukitaka Ishimoto, Hikari Matsumoto, Sakumi Nakagawa, Minako Ueda, Satoru Tsugawa

**Affiliations:** 1 Department of Mechanical Engineering, Faculty of Systems Science and Technology, Akita Prefectural University, Yurihonjo, Japan; 2 Faculty of Science and Engineering, Saga, Japan; 3 Graduate School of Life Sciences, Tohoku University, Sendai, Japan; 4 Suntory Rising Stars Encouragement Program in Life Sciences (SunRiSE)

## Abstract

Plant zygote cells exhibit tip growth, producing a hemisphere-like tip. To understand how this hemisphere-like tip shape is formed, we revisited a viscoelastic–plastic deformation model that enabled us to simultaneously evaluate the shape, stress and strain of Arabidopsis (*Arabidopsis thaliana*) zygote cells undergoing tip growth. Altering the spatial distribution of cell wall extensibility revealed that cosine-type distribution and growth in a normal direction to the surface create a stable hemisphere-like tip shape. Assuming these as constraints for cell elongation, we determined the best-fitting parameters for turgor pressure and wall extensibility to computationally reconstruct an elongating zygote that retained its hemisphere-like shape using only cell contour data, leading to the formulation of non-dimensional growth parameters. Our computational results demonstrate the different morphologies in elongating zygotes through effective non-dimensional parameters.

## Graphical abstract

**Figure:**
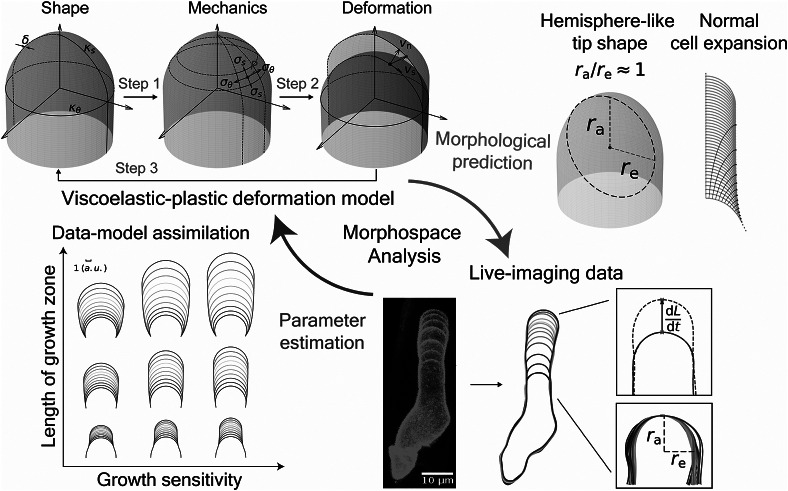

## Introduction

Growth patterns in cells are divided into two types: diffuse growth, where the entire cell surface grows and tip growth, where only the tip region grows (Kropf et al., 1998). Characteristic features of tip-growing cells include unique cell wall properties, cytoskeleton organisation and organelle activities (Rounds & Bezanilla, 2013). Since plant cell growth is thought to be controlled by cell wall biosynthesis and orientation (Green, 1962; Ledbetter & Porter 1963), quantifying cell growth patterns is important for understanding the cell wall properties behind growth, as described more precisely below. To avoid ambiguity, we define surface growth or surface elongation as extension on the cell surface and define surface point velocity as the time derivative of the displacement of the surface point. Early studies of tip growth in *Phycomyces* fungi quantified surface point velocity with tips estimated to grow at 1.2–1.4 mm per hour (Castle, 1942; Castle, 1958), while *Nitella* rhizoids were found to have a linear growth velocity of 1.7 μm/min (estimated as 0.1 mm/h) at the tip dome (Chen, 1973). During tip growth of root hairs in *Medicago truncatula* and pollen tubes in *Lilium longiflorum*, the maximal elongation zone is not located exactly at the tip but is instead located in the region slightly proximal from the tip (Shaw et al., 2000; Dumais et al., 2004; Geitmann & Dumais, 2009). These quantitative analyses indicate the importance of detailed quantification when studying tip growth in cells.

In addition to elucidating where the elongation zone is located, quantification of the direction of surface point velocity is also important. For example, imaging data from fungal hyphae with carbon particles on the cell surface revealed that some of the displacements were in directions almost perpendicular to the surface (Bartnicki-Garcia et al., 2000). That study inferred two important aspects using a mathematical model: (1) The driving force should be turgor pressure and (2) the vesicle supply centre should be consistent with the direction almost normal to the cell surface. Viscoelastic–plastic deformation models based on this pioneering mathematical study have been extensively applied to study tip growth (Goriely & Tabor, 2003; Dumais et al., 2006; Dumais, 2021). In these models, cell morphology, the mechanics on the cell surface and the deformation of the apical region are simultaneously analysed using mathematical equations. This allows the prediction of cell shape based on the history of the mechanics and deformation of the cell. These mathematical studies have prompted other studies using mechanical models (finite element methods) with modified material properties in pollen tubes (Fayant et al., 2010) and the introduction of non-dimensional parameters that characterise cell shape independently of cell size for studying tip growth (Campàs et al., 2012). Therefore, the mathematical relationships among morphology, mechanics and deformation can be used to determine the cell wall properties during cell growth.

As described above, tip growth occurs in *Nitella* rhizoids, fungal hyphae, root hairs and pollen tubes. We recently found that plant zygote cells also exhibit tip growth (Kang et al., 2023). In our previous study, we combined live imaging with the so-called normalised coordinate to show that only markers (a pollen grain expressing a sperm-specific plasma membrane) near the tip moved while other markers outside the tip region did not move. Furthermore, the elongating tip of a single zygote cell shows a characteristic hemisphere-like shape. In Arabidopsis (*Arabidopsis thaliana*) zygotes, microtubules (MTs) form a ring-like structure in the subapical region, which might support the hemisphere-like tip shape (Kimata et al., 2016). The hemisphere-like shape and MT ring are not typical in tip-growing cells of angiosperms, but similar structures are found in the tip-growing protonema of the southern maidenhair fern (*Adiantum capillus-veneris*) (Murata and Wada 1989). In this fern protonema, cellulose microfibrils are aligned in parallel to MTs, and cell division occurs. Therefore, the zygote might utilise fern-like tip growth to produce a spherical daughter cell at the tip, which develops into a globular embryo (Ueda et al., 2011). Since such an approximately hemispherical shape during elongation has also been observed in the cells of fission yeast (*Schizosaccharomyces pombe*; Abenza et al., 2015), the existence of a unifying mechanism for shaping a spherical tip needs to be investigated.

**Figure 1.**
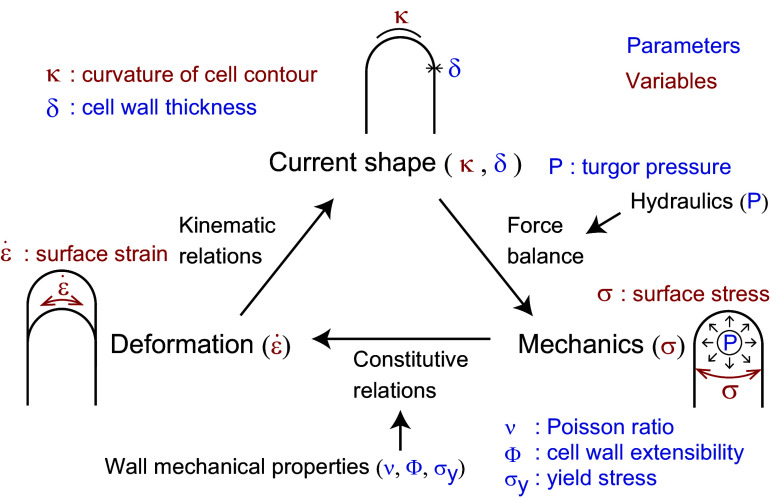
Schematic illustration of the viscoelastic–plastic deformation model. The model output is the cell contour of a tip-growing cell. By applying the hydraulics parameter (turgor pressure, 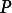 ) to the current shape with curvature 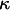 and wall thickness 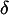 , the mechanics with stress 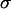 are determined. By modifying the mechanical parameters of the cell wall ( 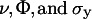 ), the deformation with strain rate 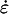 is determined.

In this study, we reimplemented the viscoelastic–plastic deformation model described by Dumais et al. (2006) to elucidate the mechanism that shapes the hemisphere-like tip. First, we obtained growth parameters in the model that matched the hemisphere-like shape described by the data. We determined that this shape results from surface point velocity almost normal to the cell surface around the tip region. Finally, we reconstructed the elongating zygote computationally using model parameters associated with turgor pressure and cell wall expansion derived from actual zygote cell contour data. Our findings shed light on the morphology and mechanics of tip growth in plant cells.

## Methods

### Viscoelastic–plastic deformation model

A viscoelastic–plastic deformation model was employed (Dumais et al., 2004; Dumais et al., 2006). This model comprises three steps (Figure 1). Step 1: The stress states in the meridional and circumferential directions ( 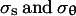 , respectively) are determined by the mechanical equilibrium of the cell with turgor pressure 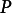 . Step 2: The strain rates in the meridional and circumferential directions ( 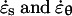 , respectively) are determined by the stresses applied on the wall and the mechanical properties of the cell wall ( 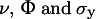 ), where 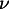 is the flow coupling (Dumais et al., 2006), 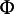 is the cell wall extensibility and 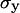 is yield stress. Step 3: The next shape is formulated using the previous cell shape and the velocity vectors ( 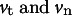 ), where 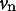 is the velocity in the direction perpendicular to the surface and 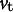 is the velocity in the meridional direction, which is one of the tangential directions.

For Step 1, the following equations were reformulated to evaluate the stress state:

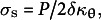




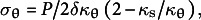

where the parameter 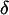 stands for cell wall thickness, and 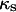 and 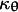 are the curvatures in the meridional and circumferential directions, respectively.

For Step 2, the following relationship was exploited:

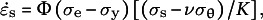




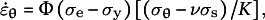

for 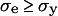 and 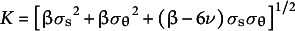 , where 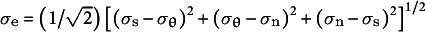 (Hill, 1998) and 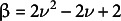 , where 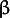 represents the anisotropy between the two directional stresses 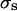 and 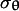 . Using 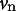 and 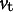 , the kinematic relations for cell shape can be evaluated as follows:

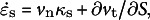




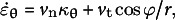

based on the cross-sectional radius 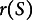 with curvilinear coordinate 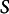 . The angle 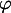 is defined as the angle between the normal vector to the surface and the axis of the cell. The normal strain rate ( 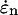 ) was assumed to always be zero, where thickening due to cell wall deposition (Houwink & Roelofsen, 1954; Kataoka, 1982) can be expressed in the following manner:

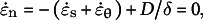

where 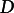 is the rate of wall deposition per unit surface area.

For Step 3, the following velocities in the normal (perpendicular) and meridional directions were obtained from the equations above:

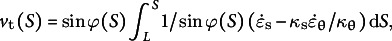




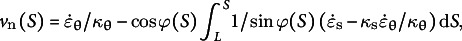

where 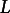 is the meridional distance between the pole and the equator. The spatial scale is based on the arbitrary unit (a.u.), which was rescaled with the typical radius scale, e.g., 5 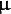 m.

### A hemispherical tip shape is sufficient for cosine-type wall extensibility

According to Green & King (1966) and under the model assumptions (Dumais et al., 2004; Dumais et al., 2006), a hemispherical cell shape is sufficient but not necessary for cosine-type wall extensibility, as described below. Considering a hemispherical cell shape with a constant radius 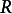 , the allometric coefficient (anisotropy rate), defined as the rate of the strain rate in the meridional direction to that in circumferential direction, can be expressed as

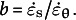



It was shown that the meridional velocity in the curvilinear direction is proportional to the 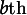 power of the sine function of the curvilinear distance 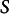 as follows:

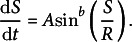



If the surface growth around the tip is approximately isotropic on the surface, characterised by 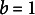 , the tip shape remains hemispherical (Green, 1969). In the derivation, assuming that the deformation of the surface is infinitesimal, the strain rate in a small meridional element 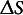 can be expressed as 

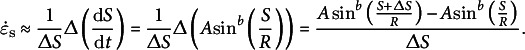



Let 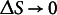 , then

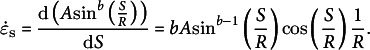

When the tip shape remains hemispherical with 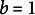 ,

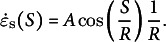



In addition, according to Dumais et al. (2004), the wall extensibility is estimated using the ratio

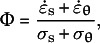

where 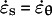 (transversely isotropic property) and 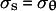 for a hemispherical tip. To substitute the equations for 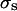 and 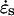 into the above expression, the following relation can be derived:

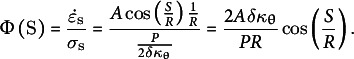



This means that a hemispherical tip geometry is a sufficient condition for cosine-type wall extensibility. Note that cosine-type wall extensibility is not sufficient for a hemispherical shape when the transversely isotropic property is not held.

## Results

### The viscoelastic–plastic deformation model can simultaneously evaluate cell shape, cell wall stresses and strain rates

**Figure 2.**
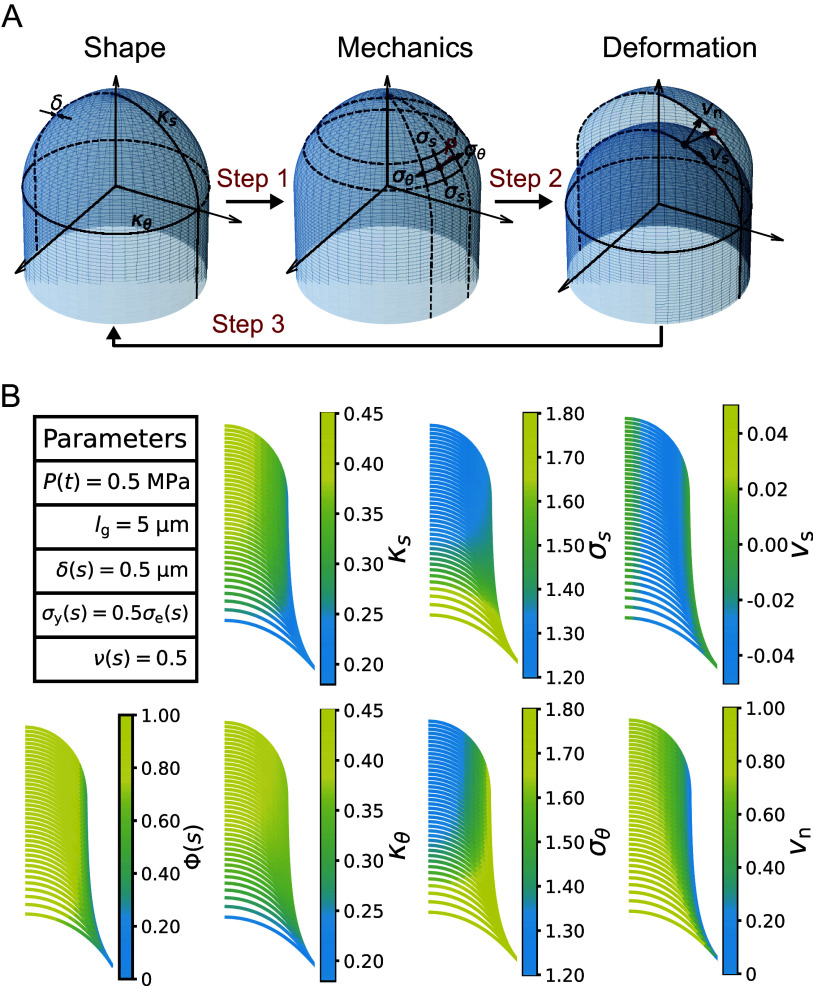
Simultaneous evaluation of the shape, mechanics and deformation of tip-growing cells. (a) Schematic representation of the model. The mechanics variable 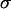 is determined from the shape data 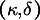 , through mechanical equilibrium with turgor pressure 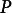 . The deformation variable 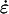 is determined from the mechanics input. (b) Evaluated shape, mechanics and deformation variables and wall extensibility.

The viscoelastic–plastic deformation model enabled us to investigate wall stresses and deformation using only the cell shape (Figure 1, Figure 2A, see details in Methods). Specifically, we used shape information (the meridional curvature 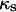 , the circumferential curvature 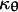 and cell wall thickness 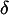 ) and turgor pressure 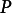 to calculate the mechanical information (the meridional stress 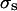 and the circumferential stress 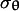 ) (Step 1). We then calculated the velocity vectors ( 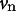 and 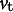 ) in the normal (perpendicular) and meridional directions associated with strain information (the meridional strain rate 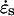 and the circumferential strain rate 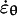 ) (Steps 2 and 3).

As the model includes the shape, mechanics and deformation of the cell at each time step, it is reasonable to describe all of these simultaneously, as shown in Figure 2B. Using the parameters in Figure 2B, we obtained the shape information 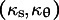 , mechanical information 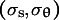 and deformational information 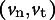 for a typical tip-growing cell with radius 1 (a.u.). This simultaneous evaluation is important because it incorporates the mechanical and deformational events simultaneously with the corresponding shape change.

### Cosine-type wall extensibility results in the formation of a hemisphere-like tip shape

**Figure 3.**
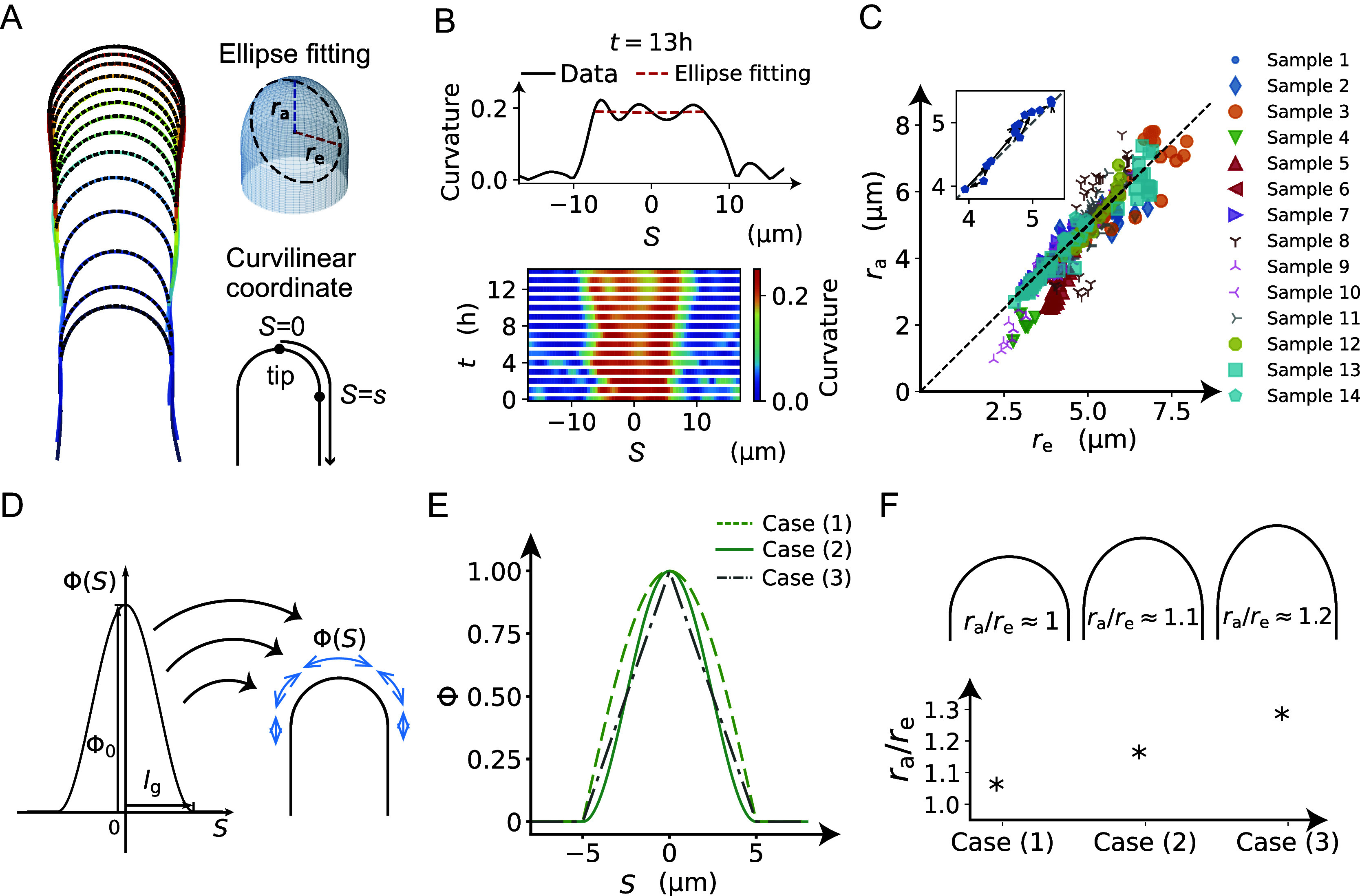
Cosine-type wall extensibility model showing a hemisphere-like shape change during cell elongation. (a) Left panel shows cell contours of a zygote with temporal colour code from blue to red with ellipse fitting (black dashed lines). Right panels show schematic illustrations of ellipse fitting and curvilinear coordinate with 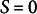 at the tip. (b) Top panel shows the curvature profile as a function of S and that from ellipse fitting (red dashed line). Bottom panel shows the spatio-temporal kymograph of the curvature. (c) Plot of 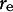 and 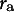 with the diagonal 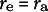 (dotted line) based on the dataset in Kang et al., 2023. (d) Spatial distribution of cell wall extensibility. Greater magnitude of cell wall extensibility indicates a high strain rate at the extension zone. (e) Three different profiles of cell wall extensibility were considered: case (1) 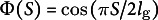 , case (2) 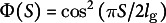 , and case (3) 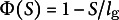 . (f) Ellipse fitting revealed that the aspect ratio of tip shape becomes close to 1.00 in the case of a cosine-type profile. The values for the three examples are 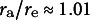 for cosine, 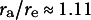 for square of cosine, and 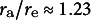 for linear function.

To further investigate the characteristic features of the model, we investigated the strain profile derived from stress input, turgor pressure and cell wall extensibility. The strain profile is defined as the curvilinear coordinate system 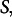 where 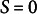 at the tip and 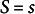 at position 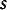 (Figure 3A). The cell wall extensibility 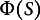 is the degree of surface growth, as presented in Figure 3D, where the magnitude at 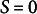 is denoted by 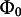 and the range of the extension zone is denoted by 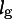 . Based on this strain profile, we explored the spatial distribution of cell wall extensibility. As the zygote maintains an approximately hemispherical shape (Figure 3A–C and Figures S1−S3), the distribution of surface growth should reflect this shape change (the current hemisphere to the next hemisphere).

The time-averaged velocities of the three example zygotes reported by Kang et al. (2023) are presented in supplementary Figure S4, where the tip has a maximum growth rate that gradually decreases as the meridional distance increases. We also confirmed that the curvature away from the tip did not change significantly over time for all the samples shown in supplementary Figure S5. This indicates that the zygote cells grow in the manner of tip growth with a hemisphere-like shape. Acknowledging that early studies (Green & King 1966; Green 1969; Dumais et al., 2004) demonstrated that the hemispherical shape is sufficient for cosine-type wall extensibility in our model, the hemispherical shape is ensured by the cosine-type wall extensibility only if the growth is transversely isotropic ( 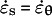 , see Methods). Therefore, we sought different types of 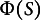 without assuming transversely isotropic growth. We employed three different formulations: case (1) 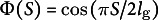 , case (2) 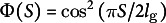 and case (3) 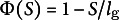 , as shown in Figure 3E. We then quantified the ellipse-fitting parameters 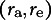 using the radial half axis 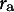 and the circumferential half axis 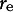 for our model (Figure 3F). By definition, the aspect ratio 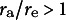 corresponds to a tapered shape, 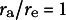 corresponds to a hemispherical shape and 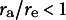 corresponds to a flattened shape. As shown in Figure 3D, the aspect ratios obtained were close to the value 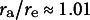 for (1), 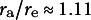 for (2) and 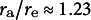 for (3). The actual data for the aspect ratio of cell shape were approximately equal to 1.00, corresponding to a hemisphere-like shape, indicating that the cosine-type wall extensibility profile is well fitted to tip-growing cells in plant zygotes.

### The hemisphere-like cell growth model exhibits a normal growth direction around the cell tip

**Figure 4.**
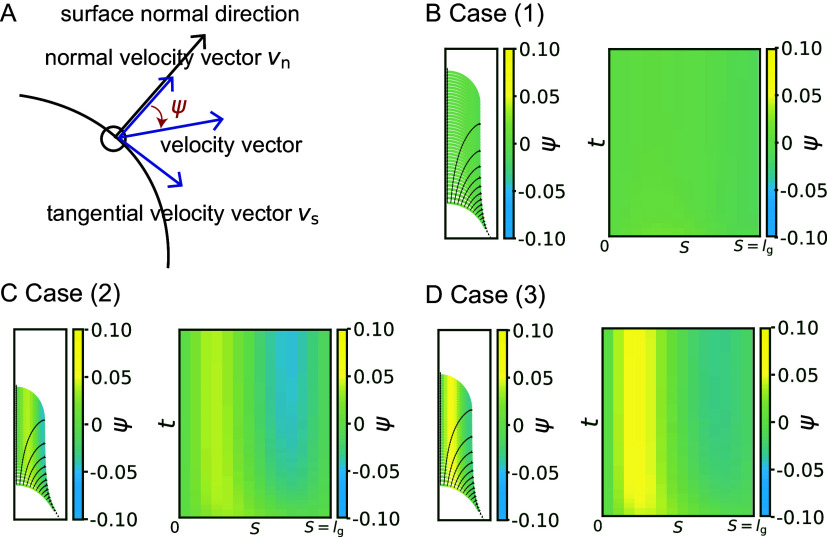
Hemispherical shape results from the normal growth direction during cell elongation. (a) Definition of the growth angle 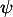 . (b–d) Growth trajectories of selected points (black lines) with colour code 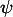 are shown in the left panel, and spatio-temporal plots of the corresponding color code 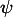 are shown in the right panel for case (1) 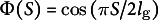 (B), case (2) 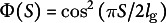 (C), and case (3) 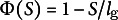 (D).

To clarify what happens to the growth direction in the model with a hemisphere-like shape, we investigated the directional angle 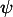 , which measures the deviated angle from the normal surface direction to the direction of surface point velocity (Figure 4A). The colour diagrams of 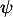 as a function of 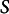 show that 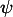 is approximately equal to 0 regardless of 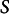 , which indicates a normal growth direction around the cell tip (Figure 4B). By contrast, the angle 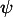 for cases (2) and (3) shows positive values in the subapical regions (Figure 4C and Figure 4D), indicating that the growth direction is not normal. Therefore, the resulting shape in cases (2) and (3) becomes more tapered. Therefore, we concluded that the cosine-type wall extensibility leads to the normal growth direction.

### An elongating zygote can be reconstructed computationally from cell contour data alone

**Figure 5.**
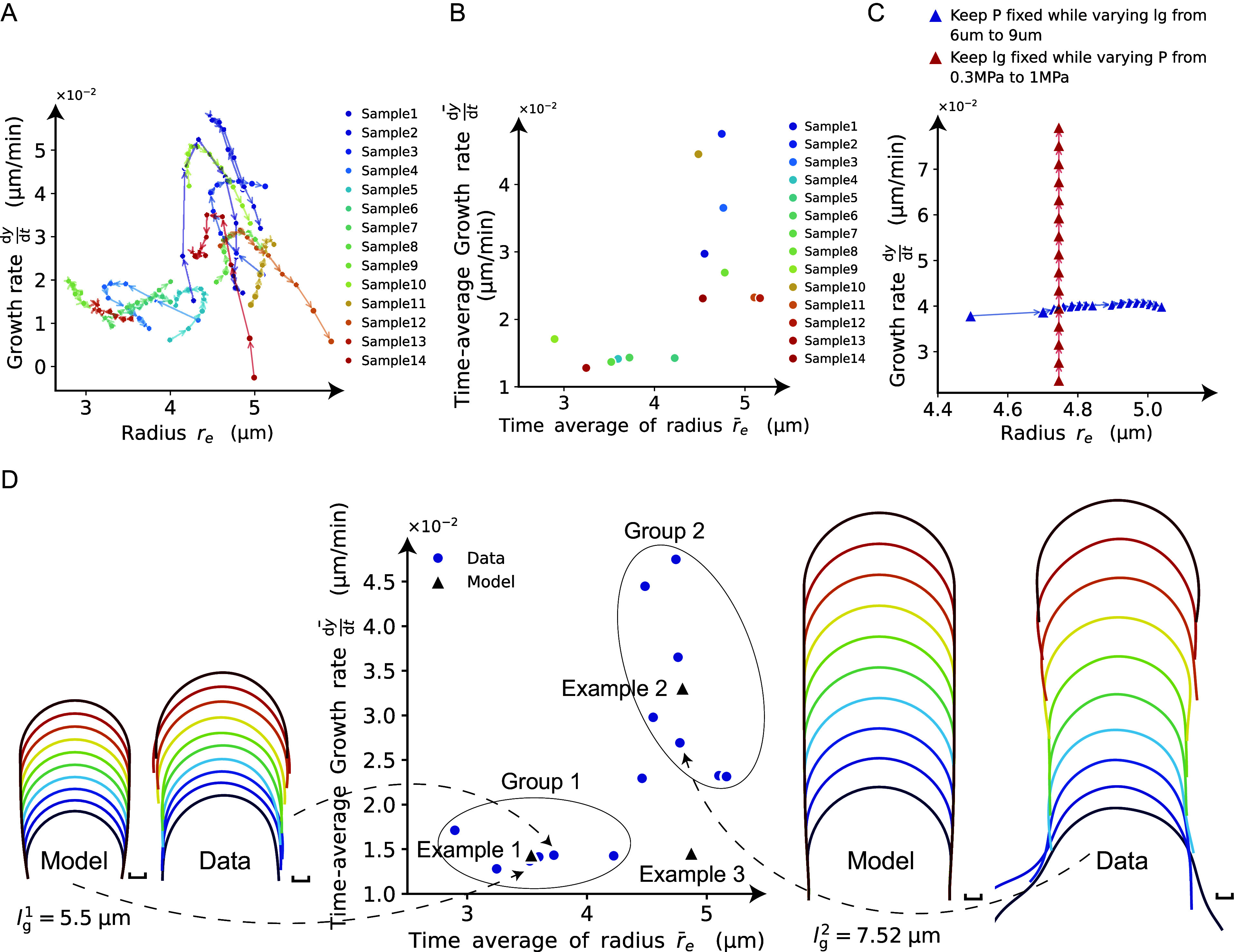
Reconstruction of model parameters using only cell contour data. (a) Morphospace analysis using the circumferential half-axis 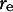 and the growth rate in the y-axis 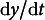 . (b) The time average of the growth rate and the radius for each sample. (c) Perturbation analysis of 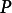 and 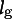 in the mechanical simulations. (d) The samples are classified into group 1 or group 2, with the sample-averaged values of 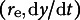 denoted by Examples 1 and 2, respectively. The left panel shows the reconstructed model for Example 1 and one data point from the contour data for group 1. The right panel shows the reconstructed model for Example 2 and one data point from the contour data for group 2.

As described above, we obtained a mathematically supported logical connection between cosine-type wall extensibility and the normal growth direction for hemisphere-like zygote shapes. The relationship points to the possibility of inferring model parameters from only cell contour data using such a constraint for wall extensibility. In our previously reported live-imaging time sequence of zygote plasma membrane markers, we obtained cell contours using the cell contour–based coordinate normalisation (CCN) method (Kang et al., 2023). Using the cell contour, we quantified the so-called morphospace 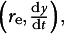 where 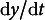 is the growth rate in the y-axis (Figure 5A). Among the previously reported samples (Kang et al., 2023), parameters were distributed in the range 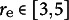 and 
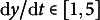
 . For the sake of simplicity, we considered the time-averaged values for each sample (Figure 5B). Based on perturbation analysis of 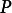 and 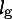 in the simulations (Figure 5C), we noticed that 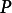 only affects the growth rate, while 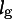 predominantly changes the radius. Therefore, we classified all the samples into group 1 or group 2 and fitted the parameter set 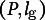 for the sample-averaged values of 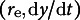 denoted by Examples 1 and 2, respectively. To further confirm what happens for intermediate parameters, we included Example 3, with almost the same growth rate as Example 1 and almost the same radius as Example 2. Using typical values for each quantity 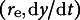 with the previously estimated range of 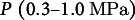 (Cosgrove, 1993; Lintilhac et al., 2000; Radotić et al., 2012) (Figure 5C), we applied an exhaustive search of 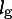 parameters that matched the data and obtained the fitted parameter 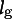 . With these parameters, we reconstructed the elongating zygote computationally (Figure 5D). Note that we assumed a constant cell wall thickness 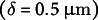 in this study based on the acknowledgement that the parameter 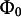 was affected by 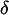 .

**Figure 6.**
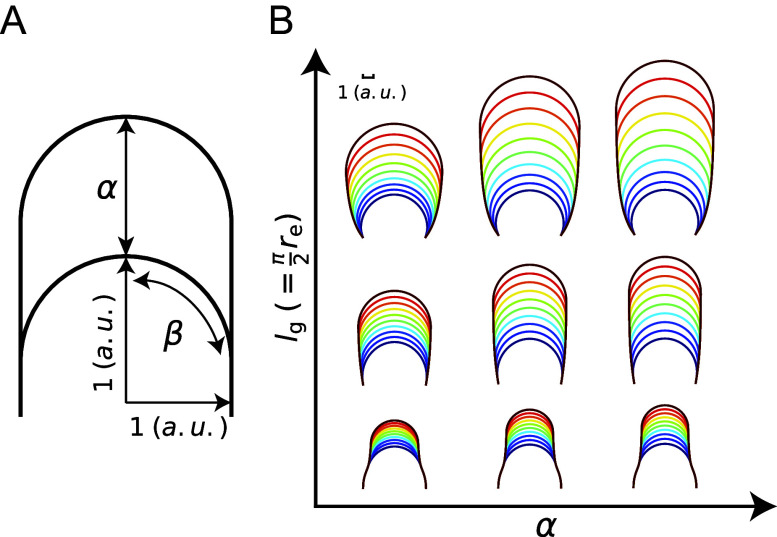
Tip-growing behavior is controlled by non-dimensional parameters 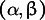 . (a) Schematic illustration of the non-dimensional parameters 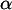 and 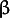 . (b) Morphospace for the elongating zygote where the stable elongating cell shape depends only on the non-dimensional parameters 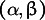 .

To summarise, we were able to reconstruct the elongating zygote computationally only from the cell contour data, with the parameter ranges inherited from the data range.

### Non-dimensional control parameters 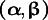 characterise tip-growing behaviour

Through the above model reconstruction, we determined effective parameters as follows. First, as the growth rate is affected by 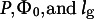 , we defined the cell growth sensitivity as

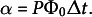



This is a non-dimensional parameter that reveals the vertical growth length (Figure 6A). As the parameter 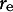 is another characteristic of the zygote cell, we can also define the constant

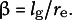



This is the other non-dimensional parameter and is the ratio between meridional growth length and hemispherical half axis length (Figure 6A). Under the hypothetical condition of the cosine-type wall extensibility profile, 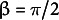 . By definition, the parameters 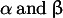 are independent of cell size; therefore, these can be used to sufficiently characterise tip-growing behaviour, as shown in Figure 6B.

## Discussion

The viscoelastic–plastic deformation model developed in this study explores three new aspects of cell elongation analysis. (1) Simultaneous evaluation of shape, mechanics and deformation, which will be a powerful tool for understanding multiple factors during cell elongation. (2) Quantitative verification of normal growth direction to produce a hemisphere-like shape. (3) Model reconstruction using only the cell contours derived from live-imaging data. Some possible future directions are summarised below.

In the field of plant mechanobiology, mechanical measurement is critical, such as in atomic force microscopy (Beauzamy et al., 2015; Tsugawa et al., 2022) and in mechanical inference from stem shape (Nakata et al., 2018). However, these methods are only applicable to the surface cells and external shapes of organs, whereas the mechanics of developmentally important cells, such as the zygote cells within the seed and lateral root primordia or vascular cells inside the root, have not yet been fully examined. Using our data–model combined method, we defined the model parameters using only the quantification of cell contours. Therefore, our method can precisely infer cellular mechanics, which cannot be determined solely by current mechanical measurement techniques, opening a new avenue for studying cellular mechanics not only in plant developmental biology but also in other multicellular systems including animal cells.

Considering the Lockhart equations (Lockhart, 1965a, 1965b), which guide the reconstruction of the plastic deformation of plant cells, the cosine-type distribution 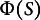 and its effect on morphology can be thought of as a spatial example of plastic deformation relating to hemisphere-like tip shape. Biological events regulating cosine-type distribution should include the heterogeneous distribution of microtubule ring structure (Kimata et al., 2016), where the microtubule-associated cell wall deposition acts as a mechanical hoop to regulate hemisphere-like tip shape. Furthermore, since our model includes viscoelastic deformation, it also considers the later-proposed viscoelastic–plastic deformation (Ortega, 1985). As revisited by Green et al. (1971), a possible first approach for reconstructing plastic deformation including viscoelasticity is to use models that take into account a threshold level of viscoelasticity. The present model precisely reflects the understanding that cell wall loosening occurs before water uptake (Cosgrove, 1985, 1987), implying that the parameter 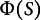 is the most important wall parameter involved in morphology, stress and strain. In addition, we identified the non-dimensional indices 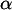 and 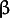 that fully characterise the dynamics of our tip-growing cells.

In summary, we developed a data-driven reconstruction method of mechanical model for studying cell elongation. This method represents a promising tool for studying the growth mechanisms and biological connections between morphology, mechanics and deformation, paving the way for understanding tip-growing cells.

## Supporting information

Kang et al. supplementary material Kang et al. supplementary material

## Data Availability

The data supporting the findings of this study are available from the author, Zichen Kang, upon a reasonable request. The code used in this study is available on GitHub (https://github.com/blues0910/A-viscoelastic-plastic-deformation-model-of-hemisphere-like-tip-growth).

## Financial statement

This work was supported by the Japan Society for the Promotion of Science [JSPS KAKENHI (JP20K15832 to S.T.), a Grant-in-Aid for Early-Career Scientists (JP22K15135 to H.M.), a Grant-in-Aid for Scientific Research on Innovative Areas (JP19H05670 and JP19H05676 to M.U.), a Grant-in-Aid for Scientific Research (B) (JP23H02494 to M.U.), International Leading Research (JP22K21352 to M.U.)], the Japan Science and Technology Agency [CREST (JPMJCR2121)], the Suntory Rising Stars Encouragement Program in Life Sciences (SunRiSE; to M.U.) and the Toray Science Foundation (20-6102 to M.U.).

## Supplementary material

The supplementary material for this article can be found at https://doi.org/10.1017/qpb.2024.13.
