# Supplementary material for: A viscoelastic–plastic deformation model of hemisphere-like tip growth in Arabidopsis zygotes
Source: Quant Plant Biol. 2024 Dec 12;5:e13. doi: 10.1017/qpb.2024.13 (PMC11706690; doi:10.1017/qpb.2024.13)
Supplement: Peer review history

# Editor report: Recommendation: A viscoelastic-plastic deformation model of hemisphere-like tip growth in Arabidopsis zygotes — R0/PR2

**DOI:** 10.1017/qpb.2024.13.pr2

**Affiliations:** John Innes Centre, United Kingdom of Great Britain and Northern Ireland

Thank you for submitting your work to QPB. Both reviewers make excellent points regarding the biology and importantly the details of the model, the simulations and the analysis that require attention. Please see their insightful comments below.

# Editor report: Decision: A viscoelastic-plastic deformation model of hemisphere-like tip growth in Arabidopsis zygotes — R0/PR3

**DOI:** 10.1017/qpb.2024.13.pr3

**Affiliations:** RDP, ENS de Lyon, France

No accompanying comment.

# Editor report: Recommendation: A viscoelastic-plastic deformation model of hemisphere-like tip growth in Arabidopsis zygotes — R1/PR5

**DOI:** 10.1017/qpb.2024.13.pr5

**Affiliations:** John Innes Centre, United Kingdom of Great Britain and Northern Ireland

Thank you for your revised manuscript. I think many aspects are now much clearer. However, it is now also apparent that are several major issues that require further attention. I think these issues need to be addressed before we can consider acceptance. I would therefore encourage you to take these latest reviewers comments into account. I’m aware that these may be substantial changes and take longer than is reasonable to expect for revisions, so I would suggest a new submission once all issues have been addressed.

# Editor report: Decision: A viscoelastic-plastic deformation model of hemisphere-like tip growth in Arabidopsis zygotes — R1/PR6

**DOI:** 10.1017/qpb.2024.13.pr6

**Affiliations:** RDP, ENS de Lyon, France

No accompanying comment.

# Editor report: Recommendation: A viscoelastic-plastic deformation model of hemisphere-like tip growth in Arabidopsis zygotes — R2/PR8

**DOI:** 10.1017/qpb.2024.13.pr8

**Affiliations:** John Innes Centre, United Kingdom of Great Britain and Northern Ireland

Thank you for your excellent revision and my apologies for the delay in the decision (I was away).

Thank you for submitting to QPB.

I looking forward to seeing your exciting work in press.

With best wishes

Richard

# Editor report: Decision: A viscoelastic-plastic deformation model of hemisphere-like tip growth in Arabidopsis zygotes — R2/PR9

**DOI:** 10.1017/qpb.2024.13.pr9

**Affiliations:** RDP, ENS de Lyon, France

No accompanying comment.
